# Correlation of renal function indicators and vascular damage in T2DM patients with normal renal function

**DOI:** 10.3389/fendo.2023.1292397

**Published:** 2023-12-18

**Authors:** Yue-Yang Zhang, Jing Gui, Bing-Xue Chen, Qin Wan

**Affiliations:** ^1^ Department of Endocrinology and Metabolism, the Affiliated Hospital of Southwest Medical University, Luzhou, China; ^2^ Metabolic Vascular Disease Key Laboratory of Sichuan Province, Luzhou, China; ^3^ Sichuan Clinical Research Center for Diabetes and Metabolism, Luzhou, China; ^4^ Sichuan Clinical Research Center for Nephropathy, Luzhou, China; ^5^ Cardiovascular and Metabolic Diseases Key Laboratory of Luzhou, Luzhou, China; ^6^ Department of Medical Imaging, Southwest Medical University, Luzhou, China

**Keywords:** type 2 diabetes mellitus, carotid atherosclerosis, renal function, eGFR, Cr

## Abstract

**Background:**

This study aimed to assess the correlation between renal function-related indices and vascular damages among patients with type 2 diabetes mellitus (T2DM) and normal renal function.

**Methods:**

We screened a cohort of eligible patients with T2DM, ultimately including 826 individuals. Utilizing multifactorial logistic regression, we conducted an in-depth analysis to explore the potential associations between renal function-related indices—specifically BUN, Cr, ALB, ACR, and eGFR—and the incidence of diabetic vascular damage. Additionally, to comprehensively understand the relationships, we employed Spearman correlation analysis to assess the connections between these indicators and the occurrence of vascular damage.

**Results:**

In this cross-sectional study of 532 patients with carotid atherosclerosis (CA), the prevalence of CA was positively correlated with Cr (53.1%, 72.3%, 68.0%, P<0.05) and negatively correlated with eGFR (71.6%, 68.5%, 53.1%, P<0.05). the higher the Cr, the higher the predominance ratio of CA (T1: reference; T2:OR. 2.166,95%CI:1.454,3.225; T3:OR:1.677, 95%CI:1.075, 2.616; P<0.05), along with an eGFR of 66.9% and 52.0% in terms of sensitivity and specificity, with a 95% CI of 0.562-0.644.

**Conclusion:**

Within our experimental sample, a noteworthy observation emerged: Creatinine (Cr) exhibited a positive correlation with the prevalence of individuals affected by carotid atherosclerosis (CA), underscoring a potential connection between Cr levels and CA incidence. Conversely, the estimated Glomerular Filtration Rate (eGFR) demonstrated a negative correlation with the occurrence of CA, implying that lower eGFR values might be associated with an increased likelihood of CA development.

## Introduction

Diabetes mellitus, a chronic and systemic metabolic disorder, stems from the intricate interplay of genetic and environmental factors over extended periods (1). Among the crucial disorders of the endocrine system, it stands as one of the most prevalent and significant (2). Statistics extracted from the 2020 Report on Nutrition and Chronic Disease Status of Chinese Residents underscore its prominence, revealing a diabetes prevalence rate of 11.9 percent among Chinese adults aged 18 and above, alongside a pre-diabetes detection rate of 35.2 percent. Notably, Type 2 diabetes exerts its most substantial impact on individuals aged 50 and above. This disease exhibits a disquieting trend toward earlier onset, prolonged duration, increased complications, amplified health risks, and augmented medical expenditures (3). The “IDF Diabetes Atlas (10th Edition),” published in 2021, forecasts that in 2021, approximately 537 million adults (aged 20-79 years) worldwide will grapple with diabetes—constituting 1 in 10 adults. This unsettling figure is anticipated to swell to 643 million by 2030 and further burgeon to 783 million by 2045. Simultaneously, as the global population is projected to grow by 20 percent within the same span, the estimated diabetes count is set to surge by 46 percent (4). These projections portend a future where more individuals are burdened by diabetes (5). Given that diabetes-associated vascular damage stands as the primary cause of mortality among T2DM patients, the imperative for enhanced predictive methodologies for these complications cannot be overstated (6).

Traditionally, risk factors linked to atherosclerosis encompass age, male gender, smoking, dyslipidemia, hypertension, and diabetes (7). Remarkably, individuals with chronic kidney disease demonstrate an elevated prevalence of atherosclerosis, a phenomenon subject to various academic propositions. These notions range from escalated oxidative stress and compromised endothelial function (8), heightened arterial rigidity (9), and compromised renal hemodynamics (10–12), to unfavorable conditions fostering plaque development and rupture (13, 14). Numerous investigations have established connections between glomerular filtration rate, serum creatinine levels, and atherosclerosis in otherwise healthy subjects before atherosclerotic onset. Intriguingly, however, no study to date has successfully identified parallels between renal function-related markers and atherosclerosis in T2DM patients with intact renal function (15).

Hence, the primary objective of this study was to meticulously assess the potential correlations between BUN, Cr, ALB, ACR, eGFR, and the occurrence of CA in patients diagnosed with T2DM, all while maintaining normal renal function.

## Methods

### Study subjects

The Division of Endocrinology and Metabolism at the Affiliated Hospital of Southwest Medical University undertook a retrospective cross-sectional investigation involving hospitalized individuals with type 2 diabetes, spanning the years 2017 to 2023. Inclusion criteria encompassed (1): Age exceeding 18 years; (2) Diagnosis by the American Diabetes Association’s “Standards of Medical Care in Diabetes” (2019 version) (16); (3) Normal renal function defined by specific parameters: BUN within the range of 2.9-7.5 mmol/L, Cr ranging from 44-133 μmmol/L, Urine Albumin (ALB) levels below 20 mg/L, albumin-to-creatinine ratio (ACR) under 30 mg/g, and eGFR exceeding 90 mL/min/1.73m². On the other hand, exclusion criteria encompassed: (1) Aberrant results in kidney function tests; (2) History of kidney disease or kidney-related surgeries; (3) Usage of kidney function-impacting medications like cyclosporine.

Data acquisition for this study was meticulously executed by healthcare professionals, employing standard questionnaires and validated equipment. Each participant furnished demographic information alongside comprehensive medical records. The categorization of participants included identifying smokers and non-smokers, as well as distinguishing current drinkers from non-drinkers. During physical examinations, mean arterial blood pressure was continuously monitored for at least 30 minutes, with three consecutive measurements taken from the right arm arterial pressure.

In the realm of ethical considerations, this study strictly adhered to the principles outlined in the Helsinki Declaration of 2013 and obtained approval from the Ethics Committee of the Affiliated Hospital of Southwest Medical University (Ethics Approval Code: 2018017) (17). Informed consent was procured from all enrolled participants.

### Calculation of eGFR


$$\text{eGFR}=170\times {\left(\text{Cr}\right)}^{-1.234}\times {\left(\text{Age}\right)}^{-0.179}\times 0.79(\text{if female})$$

### Diagnosis of vascular damage in diabetes mellitus

Carotid artery intima-media thickness (CA-IMT) measurement has established itself as a secure and replicable technique to assess atherosclerosis severity. With guidance from ultrasound physicians, the bilateral carotid arteries of all participants underwent scanning using an identical color Doppler ultrasonic diagnostic apparatus. Throughout the data collection phase, a single operator carried out the measurements. The criterion for identifying atherosclerotic plaques hinged on a local carotid artery intima-media thickness (CIMT) ≥ 1.5 mm or a local CIMT surpassing 50% of the outer surface area. A diagnosis of carotid atherosclerosis was predicated on a CIMT increase ≥ 1.0 mm and/or the presence of carotid artery plaques (18–21).

### Statistical analysis

Baseline clinical characteristics of CA patients with different genders were compared using descriptive statistics. For group comparisons, one-way analysis of variance (ANOVA) was employed for normally distributed continuous variables, the Kruskal-Wallis H test for non-normally distributed continuous variables, and the Chi-square test (X^2^ test) for categorical variables. The assessment of variables impacting diabetes vascular damages was accomplished using a Logistic regression analysis model, we additionally adjusted for age, sex, FBG, HbA1c, BMI, duration of diabetes, history of smoking, history of drinking, history of hypoglycemic drug usage, and history of insulin usage. To establish the relationship between renal function indicators and vascular damage, Spearman correlation analysis was employed. The predictive accuracy of renal function-related indicators for vascular damages was evaluated through Receiver Operating Characteristic (ROC) curves. All hypotheses were tested at a two-tailed significance level of 0.05. Risk factors were evaluated based on their odds ratio (OR) values. Forest plots were constructed using GraphPad Prism (version 9.0). The entire data analysis was conducted utilizing SPSS (version 26.0).

## Results

**Figure 1:**
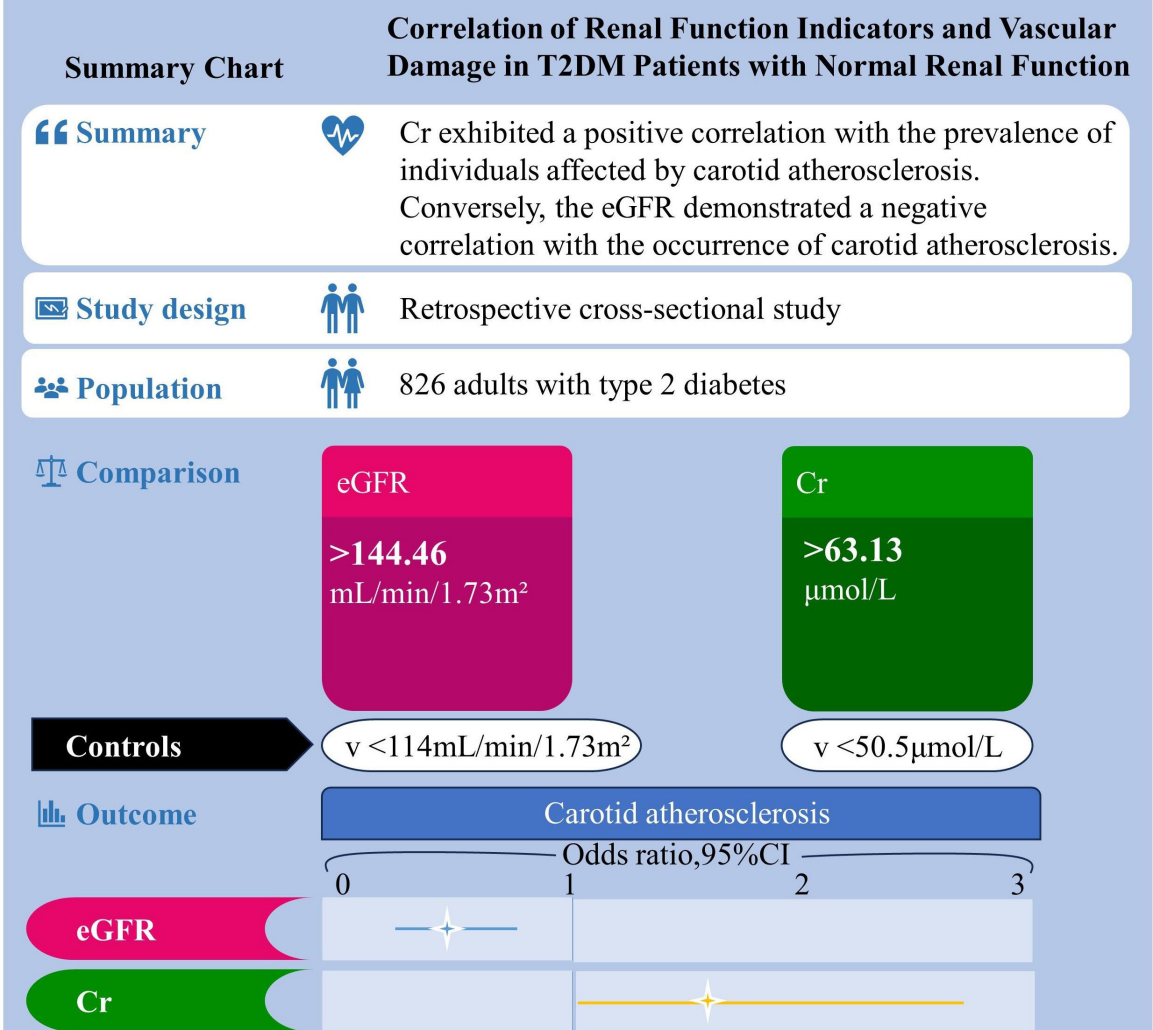
Summary chart. Correlation of renal function indicators and vascular damage in T2DM patients with normal renal function.

**Figure 1** shows a summary chart that visualizes the summary information of this article. A total of 826 T2DM patients participated in this study, comprising 440 males and 386 females. Males had an average age of 53.61 ± 10.35 years, while females averaged 58.33 ± 10.19 years. The mean duration of diabetes was 78.93 ± 76.73 months, and the study documented a total of 532 cases of cardiovascular damage. A comprehensive presentation of participants’ demographic and biochemical information is detailed in **Table 1**. Comparative analysis revealed noteworthy distinctions between males and females. Specifically, males exhibited significantly higher values in height, weight, body mass index (BMI), waist circumference (WC), diastolic blood pressure (DBP), triglycerides (TG), fasting blood glucose (FBG), alanine transaminase (ALT), blood urea nitrogen (BUN), serum creatinine (Cr), urine microalbumin (ALB), current smoking rate, and current drinking rate (all with P < 0.05). Conversely, females displayed significantly higher mean values in average age, systolic blood pressure (SBP), high-density lipoprotein cholesterol (HDL), urine albumin-to-creatinine ratio (ACR), and estimated glomerular filtration rate (eGFR) (all with P < 0.05).

**Table 1:** Clinical characteristics according to gender.

Variables	Men(N=440)	Women(N=386)	P
Age,years old	53.61 ± 10.35	58.33 ± 10.19	<0.001^*^
Height,cm	166.44 ± 5.91	154.46 ± 10.17	<0.001^*^
Weight,kg	68.99 ± 11.05	58.15 ± 10.17	<0.001^*^
BMI,Kg/m^2^	24.85 ± 3.38	24.32 ± 3.62	0.032^*^
WC,cm	88.36 ± 9.69	83.35 ± 9.62	<0.001^*^
SBP,mmHg	127.65 ± 16.63	131.66 ± 18.05	0.001^*^
DBP,mmHg	77.96 ± 10.40	75.42 ± 9.64	<0.001^*^
TG,mmol/L	2.45 ± 2.31	2.04 ± 1.89	0.006^*^
TC,mmol/L	4.57 ± 1.24	4.70 ± 1.10	0.125
LDL,mmol/L	2.70 ± 0.93	2.80 ± 0.93	0.128
HDL,mmol/L	1.09 ± 0.33	1.25 ± 0.35	<0.001^*^
FBG,mmol/L	9.20 ± 3.38	8.54 ± 3.09	0.006^*^
HbA1c,%	9.85 ± 2.52	9.37 ± 2.53	0.008^*^
ALT,mmol/L	35.42 ± 48.86	27.52 ± 23.3	0.004^*^
AST,mmol/L	26.13 ± 33.49	24.14 ± 18.39	0.299
BUN,mmol/L	5.43 ± 1.16	5.20 ± 1.13	0.004^*^
Cr,μmol/L	65.91 ± 14.00	50.39 ± 12.09	<0.001^*^
ALB,mg/L	6.45 ± 6.02	5.34 ± 5.52	0.006^*^
ACR,mg/g	10.32 ± 6.44	11.82 ± 6.80	0.001^*^
eGFR,mL/min/1.73m²	127.55 ± 33.26	143.60 ± 84.50	<0.001^*^
Duration of diabetes,mouth	74.01 ± 73.03	84.74 ± 80.61	0.057
Current Drinking(No/Yes)	179/261	368/18	<0.001^*^
Current Smoking(No/Yes)	199/241	379/7	<0.001^*^
Hypoglycemic Drugs(No/Yes)	182/258	140/246	0.135
Insulin(No/Yes)	331/109	303/83	0.267

The values were expressed as the mean ± SD, n. BMI, body mass index; WC, waist circumference; SBP, systolic blood pressure; DBP, diastolic blood pressure; TG, triacylglycerol; TC, total cholesterol; LDL, low-density lipoprotein cholesterol; HDL, high-density lipoprotein cholesterol; FBG, fasting blood glucose; HbA1c, hemoglobin A1c; ALT, alanine aminotransferase; AST, aspartate aminotransferase; BUN, blood urea nitrogen; Cr, Creatinine; ALB, urine albumin; ACR, albumin-to-creatinine ratio; eGFR, estimated glomerular filtration rate; *P<0.05.

**Table 2** illustrates the distribution of CA across the tertiles of BUN, Cr, ALB, ACR, and eGFR. A direct correlation was identified between the prevalence of CA and Cr(53.1%,72.3%,68.0%, P<0.05) and BUN(59.7%,65.5%,68.1%, P<0.05)levels, whereas an inverse relationship was observed with eGFR(71.6%, 68.5%,53.1%, P<0.05). Notably, the prevalence of CA remained nearly consistent across all tertiles of ALB, and ACR.

**Table 2:** Prevalence of CA in different renal function indicator tertiles.

Events	Carotid atherosclerosis	P-value
BUN		0.039^*^
T1(<4.79)	166(59.7%)	
T2(4.79˜5.90)	180(65.5%)	
T3(>5.90)	186(68.1%)	
Cr		<0.001^*^
T1(<50.50)	147(53.1%)	
T2(50.50˜63.13)	198(72.3%)	
T3(>63.13)	187(68.0%)	
ALB		0.129
T1(<1.10)	181(65.6%)	
T2(1.10˜8.10)	189(68.2%)	
T3(>8.10)	162(59.3%)	
ACR		0.588
T1(<7.20)	183(68.3%)	
T2(7.20˜12.90)	174(62.8%)	
T3(>12.90)	175(64.1%)	
eGFR		<0.001^*^
T1(<114.10)	197(71.6%)	
T2(114.10˜144.46)	189(68.5%)	
T3(>144.46)	146(53.1%)	

The values were expressed as n (%). *P<0.05.

Odds ratios for CA were computed through a multivariate regression model, as depicted in **Table 3**. In Model 2, after gender adjustment, higher Cr levels were linked with amplified odds ratios for CA(T1: reference; T2:OR:2.158,95%CI: 1.473, 3.162; T3: or:1.696, 95%CI: 1.122, 2.565; P<0.05). This pattern persisted predominantly in Model 3, even after considering the influence of other potentially confounding variables (T1:reference; T2:OR:2.166,95%CI:1.454,3.225; T3:OR:1.677,95%CI:1.075, 2.616; P<0.05). A direct correlation emerged between Cr levels and CA risk, while eGFR displayed an inverse pattern. Regrettably, no discernible correlations materialized between BUN, ALB, ACR, and the prevalence of CA.

**Table 3:** Corrected OR and 95% CI for tertiles of renal function indicators.

Events	Model 1	P-value	Model 2	P-value	Model 3	P-value
BUN						
T1	1	0.108	1	0.166	1	0.405
T2	1.278(0.905,1.806)	0.163	1.248(0.882,1.766)	0.212	1.172(0.822,1.672)	0.381
T3	1.442(1.017,2.046)	0.040	1.394(0.980,1.982)	0.064	1.274(0.888,1.826)	0.188
Cr						
T1	1	<0.001^*^	1	<0.001^*^	1	0.001^*^
T2	2.304(1.616,3.284)	<0.001^*^	2.158(1.473,3.162)	<0.001^*^	2.166(1.454,3.225)	<0.001^*^
T3	1.879(1.329,2.657)	<0.001^*^	1.696(1.122,2.565)	0.012^*^	1.677(1.075,2.616)	0.023^*^
ALB						
T1	1	0.083	1	0.044	1	0.064
T2	1.127(0.791,1.607)	0.508	1.130(0.791,1.614)	0.502	1.049(0.729,1.510)	0.797
T3	0.766(0.542,1.083)	0.131	0.731(0.515,1.038)	0.080	0.707(0.494,1.013)	0.059
ACR						
T1	1	0.687	1	0.736	1	0.781
T2	0.859(0.606,1.217)	0.391	0.873(0.615,1.240)	0.449	0.880(0.616,1.225)	0.493
T3	0.907(0.639,1.290)	0.588	0.961(0.674,1.371)	0.827	0.936(0.652,1.344)	0.720
eGFR						
T1	1	<0.001^*^	1	<0.001^*^	1	<0.001^*^
T2	0.860(0.597,1.239)	0.419	0.865(0.600,1.248)	0.439	0.861(0.590,1.255)	0.436
T3	0.448(0.315,0.638)	<0.001^*^	0.474(0.332,0.678)	<0.001^*^	0.467(0.317,0.689)	<0.001^*^

Model 1: unadjusted; Model 2: adjusted for sex; Model 3: adjusted for sex, FBG, HbA1c, BMI, duration of diabetes, current smoking, current drinking, hypoglycemic drugs, insulin. OR, odds ratio; CI, confidence interval. *P<0.05.

As depicted in **Table 4**, the connection between renal function indicators and CA in T2DM patients with normal kidney function was established through Spearman correlation analysis. This analysis unveiled a positive correlation between Cr(rs=0.144, P<0.001) and BUN(rs=0.068, P=0.049) with CA, while eGFR(rs=-0.170, P<0.001) exhibited a negative correlation. However, no substantial association was discerned between CA and ALB or ACR.

**Table 4:** Association of renal function indicators with carotid atherosclerosis.

Events	CA, rs	P
BUN	0.068	0.049^*^
Cr	0.144	<0.001^*^
ALB	-0.050	0.153
ACR	-0.032	0.353
eGFR	-0.170	<0.001^*^

BUN, blood urea nitrogen; Cr, Creatinine; ALB, urine albumin; ACR, albumin-to-creatinine ratio; eGFR, estimated glomerular filtration rate; CA, carotid atherosclerosis; rs, Spearman’s correlation coefficient. *P<0.05.

**Figure 2:**
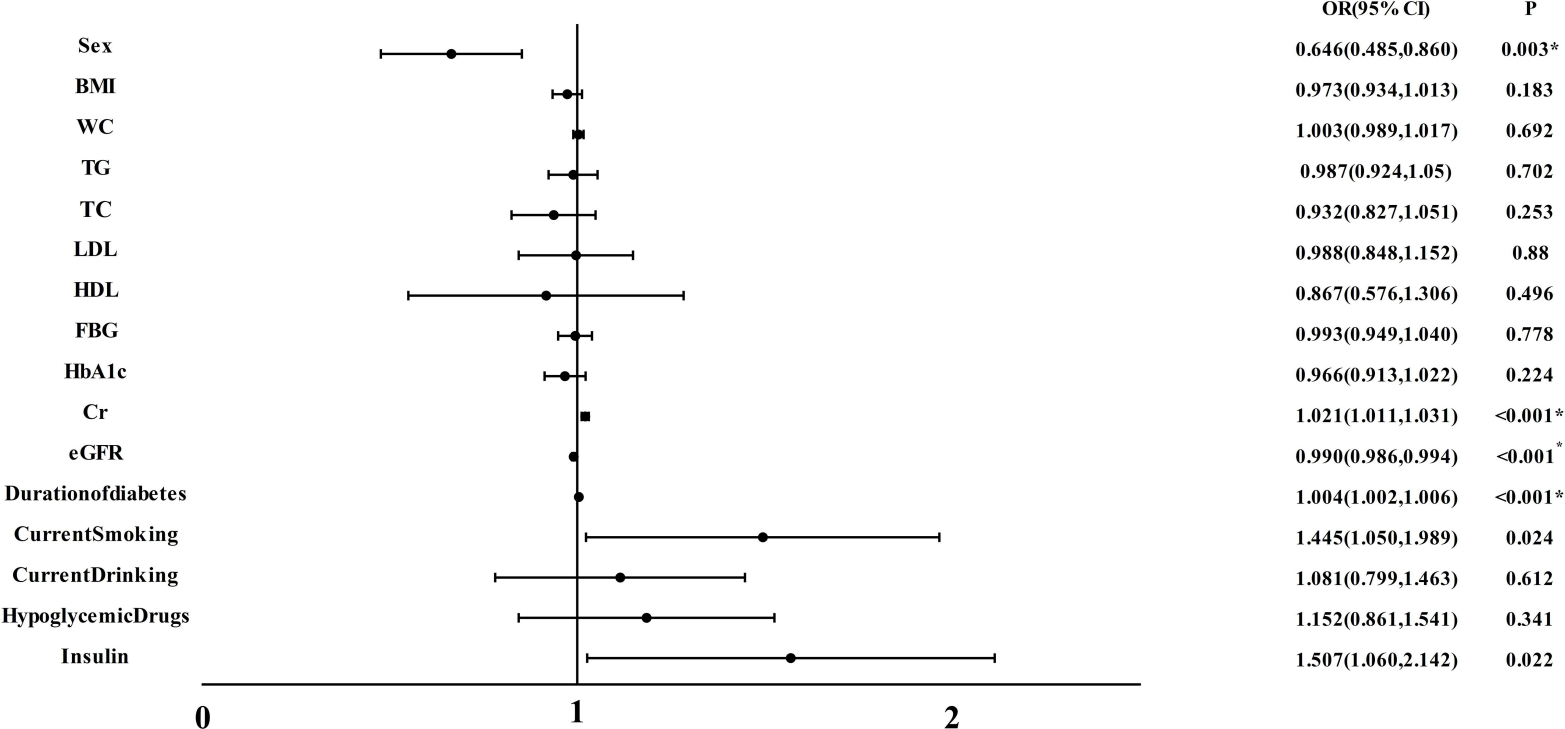
Multiple regression analysis of variables independently associated with CA in all participants. BMI, body mass index; WC, waist circumference; TG, triacylglycerol; TC, total cholesterol; LDL, low-density lipoprotein cholesterol; HDL, high-density lipoprotein cholesterol; FBG, fasting blood glucose; HbA1c, hemoglobin A1c; Cr, Creatinine; eGFR, estimated glomerular filtration rate; CA, carotid atherosclerosis. *P<0.05.

As illustrated in **Figure 2**, we conducted a multivariable regression analysis on variables that remain independent of diabetes-related vascular damage. The forest plot provided a visual representation, showcasing Cr(OR:1.021, 95%CI:1.011,1.031) and Duration of diabetes(OR:1.004, 95%CI:1.0020,1.006) as the notable risk factor for CA(all P < 0.001). Moreover, it was observed that eGFR(OR: 0.990, 95% CI:0.986, 0.994, P<0.001) played a protective role against CA development.

**Figure 3:**
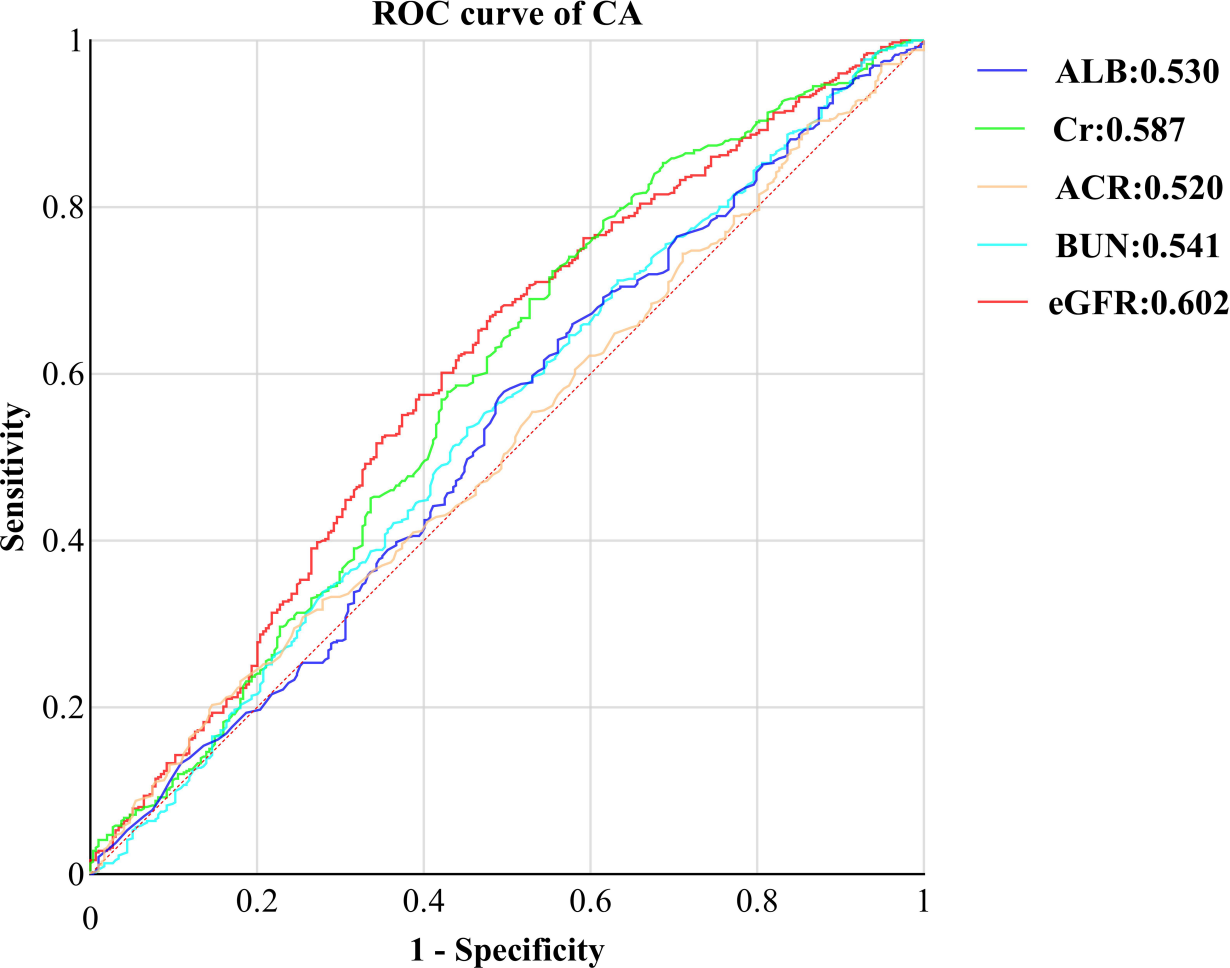
ROC curve of kidney function indicators CA in T2DM with normal kidney function. ROC, receiver operating characteristic; CA, carotid atherosclerosis; ALB, urine albumin; Cr, Creatinine; ACR, albumin-to-creatinine ration; BUN, blood urea nitrogen; eGFR, estimated glomerular filtration rate.

Finally, we evaluated the diagnostic efficacy of renal function-related indicators for CA through Receiver Operating Characteristic (ROC) curves (**Figure 3**). Remarkably, eGFR emerged as the most accurate indicator, boasting the highest Area Under the Curve (AUC) of 0.062 (95% CI: 0.562, 0.644, P<0.001). This was succeeded by Cr(AUC: 0.587, 95% CI: 0.54, 0628, P<0.001), BUN(AUC: 0.541, 95% CI:0.500, 0.583, P=0.049), ALB(AUC:0.530, 95% CI:0.488, 0.572, p=0.154), and ACR(AUC:0.520, 95% CI:0.479, 0.560, P=0.352). By utilizing the Youden index, we determined the optimal cutoff value for eGFR to be 138.2. The sensitivity of eGFR reached 66.9%, accompanied by a specificity of 52.0%.

## Discussion

This cross-sectional study involved 826 T2DM patients and sought to investigate the interplay between CA prevalence and renal function-related indicators, specifically BUN, Cr, ALB, ACR, and eGFR, in T2DM patients possessing normal kidney function. Data analysis outcomes unveiled an escalating CA prevalence as Cr quartiles ascended (P < 0.05), while a distinct reduction in CA patients was discerned among the upper eGFR quartiles (P < 0.05). Following the adjustment for confounding factors, including gender, a positive correlation emerged between Cr and CA (P < 0.05), while eGFR exhibited a negative association with CA (P < 0.05).

Collectively, kidney function demonstrated an adverse connection with CA, remaining unaffected by recognized risk factors such as age, gender, blood pressure, serum LDL cholesterol, and blood glucose. Remarkably, eGFR showcased the most robust performance in evaluating the correlation between renal function indicators and CA prevalence, closely trailed by Cr. Regrettably, up to this point, significant correlations between BUN, ALB, ACR, and CA prevalence have yet to be observed in T2DM patients boasting normal kidney function.

Furthermore, an intriguing yet perplexing observation surfaced during our analysis. While employing Spearman correlation analysis to discern the interrelation between renal function-related indicators and CA in T2DM patients with normal kidney function, BUN(rs=0.068, P=0.049) seemed to hint at a plausible positive correlation with CA occurrence. However, the outcomes derived from applying a multivariable regression model failed to demonstrate a significant association between the two. This discrepancy could potentially be attributed to the relatively modest size of our dataset or even potential limitations within our research methodology.

Currently, numerous research groups have delved into the intricate relationship between renal function and the onset of CA within diverse clinical contexts. For instance, Silvio Buscemi et al. (15) uncovered a distinct link between GFR and CA in individuals without renal insufficiency, substantiating a consistent negative correlation between renal function and CA, which aligns with our findings. Moreover, in patients grappling with chronic kidney failure, there has been validated affirmation of a closely intertwined association between compromised renal function and atherosclerosis. Multi-ethnic investigations involving a substantial cohort of Chronic Kidney Disease (CKD) patients have consistently reported a robust correlation between carotid intima-media thickness (c-IMT) and GFR, irrespective of patients’ metabolic conditions (22–24). Notably, even during the incipient stages of chronic kidney failure, the prevalence of CA is augmented (25). Certain inquiries have spotlighted a nexus between atherosclerosis and diminishing GFR or Cr levels, irrespective of the patient’s renal function status (12, 26)

Consequently, amalgamating the insights from our study with the existing body of research, a tentative inference can be drawn: sustaining GFR in proximity to the lower boundary of the normal range and maintaining Cr within the upper threshold of normal across the long term might potentially foster systemic atherosclerosis, exacerbate atherosclerotic damage, and potentially yield unfavorable consequences for kidney health. Furthermore, we have observed that the optimal eGFR threshold, calculated using the Youden index, is 138.2 ml/min/m^2^. This value suggests that patients may be experiencing a state of hyperfiltration. While some studies have indicated an upward trend in eGFR with the progression of diabetes, it is worth noting that a high eGFR is recognized as one of the risk factors for diabetic kidney disease (27). Notably, the early onset of high filtration in diabetes is associated with more severe kidney damage in the advanced stages of the disease (28–31). However, the determination of the factors contributing to renal hyperfiltration involves intricate physiological and pathological mechanisms, making it challenging to pinpoint whether this state results from physiological or pathological factors. Additionally, there is currently a shortage of relevant research exploring the relationship between kidney function and vascular damage in diabetes patients who are experiencing hyperfiltration. To a certain extent, our study addresses this research gap. Of course, it’s important to acknowledge a counter perspective presented by David Leander Rimmele et al., asserting that CA is independently associated with higher levels of NT-proBNP, through common risk factors and NT-proBNP with AF, and not with renal function (32).

Unlike our study and that of Silvio Buscemi, the divergent findings in the research by David Leander Rimmele et al. could plausibly emanate from varying population characteristics across different geographical regions or differences in methodological approaches.

Moreover, given that it stands as one of the most prevalent complications of Type 2 Diabetes Mellitus (T2DM), the significance of early intervention in addressing CA cannot be overstated, as it profoundly impacts patients’ quality of life. Numerous studies have delved into the intricate web of associations between diverse indicators in T2DM patients and CA. For instance, the work of Jie Lin et al. (33) underscored a discernible correlation between thyroid-related hormones, diabetic peripheral neuropathy, and CA. Concurrently, Chenxi Wang et al. brought to the forefront the independent links between liver fat content index, fatty liver index, and CA within the landscape of T2DM. These indices could potentially serve as straightforward and invaluable markers, facilitating the evaluation of diabetic macrovascular damages and their progression (34).

The underlying biological mechanisms that tether Cr, GFR, and carotid atherosclerosis may be entwined with more profound factors. According to the perspective put forth by Kazuyuki Yahagi et al. (25), the pivotal driving forces behind diabetic atherosclerosis encompass a panorama of elements including oxidative stress, endothelial dysfunction, alterations in mineral metabolism, an overabundance of inflammatory cytokines, and even the mobilization of bone progenitor cells into the circulation. Nevertheless, it’s important to acknowledge that due to the constrained exploration of these factors within our study, a comprehensive elucidation remains elusive, thereby underscoring the necessity for further animal studies to plumb the depths of this intricate subject.

Typically, other researchers tend to focus on exploring the correlation between a single indicator and CA. In contrast, this study adopted an innovative approach, delving into the correlation between five renal function indicators – namely eGFR, Cr, BUN, ALB, and ACR – and CA in patients with T2DM. This distinct methodology yields more comprehensive insights that hold immense value for clinical practitioners managing T2DM patients, regardless of their CA status. However, it’s important to acknowledge the limitations inherent in this study. Chief among them is the relatively modest sample size, which could potentially introduce a degree of imprecision into the findings. Equally important, our study has certain limitations in diagnosing vascular damage in diabetes. According to the new expert consensus (35), flow-mediated dilation (FMD) and arterial stiffness are considered more valuable for assessing vascular damage, with FMD often regarded as the gold standard. However, in this study, we employed IMT as the diagnostic indicator for vascular damage, which is not widely recognized. This choice was made because FMD is not commonly used for diagnosing vascular damage in our study region, which may introduce some degree of error in the study results. Furthermore, the cross-sectional design employed in this research falls short of establishing definitive causality and fails to provide an intricate dissection of the mechanisms underpinning the connection between eGFR, Cr, and CA in T2DM patients. To substantiate the potential causal link between eGFR, Cr, and CA as unearthed in this study, subsequent longitudinal investigations are imperative.

## Conclusions

In the context of T2DM patients possessing normal renal function, it becomes evident that those with lower eGFR levels and higher Cr levels are predisposed to a heightened likelihood of developing CA. Recognizing that elevated Cr and diminished eGFR serve as risk factors for CA, it’s noteworthy that even when patients’ eGFR and Cr values fall within the normal range, proactively managing and maintaining optimal eGFR and Cr levels within the T2DM population could yield substantial benefits in terms of CA prevention.

## Data availability statement

The raw data supporting the conclusions of this article will be made available by the authors, without undue reservation.

## Ethics statement

The studies involving humans were approved by the ethics committee of the Affiliated Hospital of Southwest Medical University. The studies were conducted in accordance with the local legislation and institutional requirements. The participants provided their written informed consent to participate in this study.

## Author contributions

Y-YZ: Writing – original draft. JG: Writing – original draft. B-XC: Data curation, Writing – original draft. QW: Writing – review & editing.
